# Characterization of Heart Diseases per Single Lead Using ECG Images and CNN-2D †

**DOI:** 10.3390/s24113485

**Published:** 2024-05-28

**Authors:** Lerina Aversano, Mario Luca Bernardi, Marta Cimitile, Debora Montano, Riccardo Pecori

**Affiliations:** 1 Department of Agricultural Science, Food, Natural Resources and Engineering, University of Foggia, 71122 Foggia, FG, Italy; 2 Department of Engineering, University of Sannio, 82100 Benevento, BN, Italy; bernardi@unisannio.it; 3 Department of Law and Digital Society, Unitelma Sapienza University, 00161 Rome, RM, Italy; marta.cimitile@unitelmasapienza.it; 4 CeRICT scrl, Regional Center Information Communication Technology, 82100 Benevento, BN, Italy; 5 Institute of Materials for Electronics and Magnetism, National Research Council of Italy, 43124 Parma, PR, Italy; 6 SMARTEST Research Centre, eCampus University, 22060 Novedrate, CO, Italy

**Keywords:** convolutional neural networks, electrode characterization, electrocardiogram (ECG) image classification, image feature extraction, heart disease

## Abstract

Cardiopathy has become one of the predominant global causes of death. The timely identification of different types of heart diseases significantly diminishes mortality risk and enhances the efficacy of treatment. However, fast and efficient recognition necessitates continuous monitoring, encompassing not only specific clinical conditions but also diverse lifestyles. Consequently, an increasing number of studies are striving to automate and progress in the identification of different cardiopathies. Notably, the assessment of electrocardiograms (ECGs) is crucial, given that it serves as the initial diagnostic test for patients, proving to be both the simplest and the most cost-effective tool. This research employs a customized architecture of Convolutional Neural Network (CNN) to forecast heart diseases by analyzing the images of both three bands of electrodes and of each single electrode signal of the ECG derived from four distinct patient categories, representing three heart-related conditions as well as a spectrum of healthy controls. The analyses are conducted on a real dataset, providing noteworthy performance (recall greater than 80% for the majority of the considered diseases and sometimes even equal to 100%) as well as a certain degree of interpretability thanks to the understanding of the importance a band of electrodes or even a single ECG electrode can have in detecting a specific heart-related pathology.

## 1. Introduction

**Figure 1:**
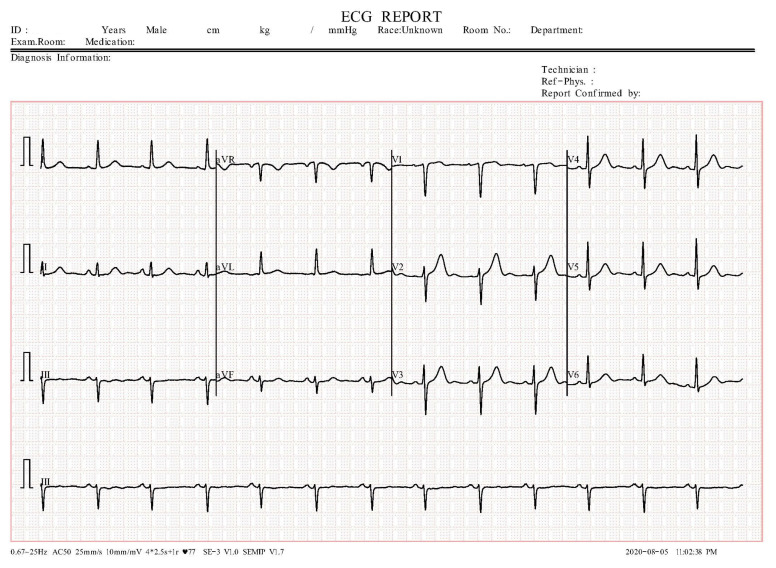
Sample of an ECG image we considered, wherein the twelve signals of an ECG are grouped into three bands, each one composed of four slices of different electric signals.

Cardiac problems constitute the most common causes of hospitalization, disability, and mortality. As a result, anticipating the course of such illnesses is very important. The electrocardiogram (ECG), a visual representation of the electrical activity of the heart throughout its function, is used to identify the majority of heart disorders. ECG is the most widely utilized diagnostic instrument in hospitals because it is the most simple to perform and the cheapest one. The process entails using a device to capture and graphically report the cardiac rhythm as well as the electrical activity of the heart. To record an ECG, 12 electrodes are usually placed on the surface of the body to detect voltage changes, which are then recorded in an ECG picture, where the resulting signal may be seen and quantified (Figure 1). An ECG picture specifically records the bio-electrical activity of the cardiac system whenever a heart illness develops, some or all of the signals given by the electrodes change from their normal condition [1]. ECG is usually preferred rather than other diagnostic tests, such as cardiac magnetic resonance, diagnostic coronary angiography, or myocardial scintigraphy, for several reasons: (i) ECG examination takes only a few minutes, (ii) the report is delivered in a maximum of half an hour, (iii) it can be performed at any age, and (iv) it is non-invasive. For all of these reasons, ECG is relevant in heart disease scientific studies and it is extensively explored, mainly for ‘arrhythmias’, a collection of irregular cardiac impulses [2]. Additionally, ECG signals are similar in patients with comparable cardiac disorders, making it feasible to forecast heart ailments by evaluating ECG signal patterns. For example, if the morphological pattern of an ECG signal with unknown features mimics that of an ECG signal with a known arrhythmia, the signal is presumed to come from a patient with the same type of arrhythmia. Moreover, it is often crucial to detect irregular heart conditions early, in order to diagnose heart problems and prevent sudden cardiac death; thus, automated heart disease detection is sometimes preferable to traditional approaches, which require laborious and time-consuming observations of ECG morphological features. Indeed, an effective algorithm could easily facilitate the automated detection of cardiac diseases and categorize ECG pictures with unknown features based on how similar they are to signals with known characteristics.

The research presented in this paper is an extension of the preliminary one presented in [3] and employs a novel analysis based on the single electrodes of the 12-lead ECG image. Furthermore, this approach allows one to understand which electrodes are the best ones to find out the different heart diseases we studied. Our approach exploits two-dimensional Convolutional Neural Networks (CNN-2D) to automate the diagnosis of cardiac abnormalities, yielding significant and unexpected findings. More in detail, the most relevant contributions of this work are the following:

- the usage of ECG images to extract the features related to the 12 ECG signals to predict heart diseases, with very appreciable performance results, especially in terms of recall, the most important metric in clinical analyses;
- the identification of the electrodes that are most useful in the classification of each different heart disease.

Hereinafter, the article is structured as follows: Section 2 provides a summary of the existing literature on the studied matter, while Section 3 introduces and discusses the deep neural network model we used (Section 3.1), describes the heart diseases we studied in this work (Section 3.2), and explains the process to record an ECG (Section 3.3); Section 4 describes the approach used to create the datasets that are described in Section 4.1, while all the characteristics of the experiment we carried out in this work are in Section 4.2, and the methodology for validating the results is explained in Section 4.3. Section 5 highlights all the outcomes we achieved as a result of this study, Section 6 discusses the main implications as well as the limitations of our findings, and, lastly, Section 7 highlights the conclusions reached by the carried out experiments together with future research perspectives.

## 2. Related Studies

In the last decades, ECG signals have aroused particular interest in the recent literature, especially concerning the automatic detection of heart diseases [4,5]. The studies in the state of the art show that there are two main methodologies for the construction of a heart disease prediction system: (i) the analysis of the parameters derived from the heartbeat and (ii) the approaches based on classification algorithms [6,7,8]. In relation to the studies on the parameters, various characteristics can be extracted from an ECG: morphological characteristics, the wavelet transform, the Fourier transform, and even the statistical parameters. Xu et al. [6] proposed a dual approach with consequential modules: the first module is based on feature extraction from heartbeat signals, followed by a classification of the heartbeat using an Extreme Learning Machine (ELM), a single-hidden layer feed-forward network. Singh et al. [7] compare several machine learning (ML) and deep learning (DL) classifiers, using time series sequences of ECGs. They precisely apply Support Vector Machine (SVM) and Bi-directional Long Short Term Memory (BiLSTM) networks to ECG data and parameters derived utilizing Maximal Overlap Discrete Wavelet Transform analysis (MODWT). Their approach shows that using DL techniques is preferable to ML because it provides much more accurate results. Furthermore, concerning the classification algorithms to predict arrhythmias, the majority of the considered studies are based on ML and DL [6,7,8,9,10,11]. These techniques are particularly suitable not only for the prediction of heart diseases, but also for difficult-to-diagnose diseases such as Parkinson’s, thyroid diseases, and cancer. DL has achieved excellent results in the detection and classification of medical images, such as ECG ones [12]. In particular, CNNs are the most implemented networks for the classification of images. A CNN is widely recognized as a state-of-the-art solution for detecting and categorizing cardiac signals, and it has been investigated in several forms, including one-dimensional (CNN-1D), two-dimensional (CNN-2D), and/or a combination of both [9,10,11]. Noman et al. [10] created a framework for immediate feature learning of raw cardiac signals based on TF-ECNN, a hybrid combination of CNN-1D, CNN-2D, and bi-dimensional time-frequency (TF) feature maps. To evaluate their approach, they also categorized the beats with more standard classifiers such as an SVM, an ensemble model of a Decision Tree, and a Hidden Markov model.

Huang et al. [13] transformed five types of heartbeats into time-frequency spectrograms for training a deep CNN-2D to recognize several types of arrhythmia. They compared the suggested model with a typical CNN-1D model to evaluate their performance. The CNN-2D significantly outperforms the CNN-1D algorithm in terms of accuracy. To categorize the ECGs, Ji et al. [14] transcribed 1D signals into 2D images, using faster R-CNN, a convolutional network composed of a ZF network that generates the feature map used as input of a second network, an RPN network that creates the final input for the fast R-CNN network that finally gives the desired outputs. The complete architecture of the faster R-CNN used by the authors counts 16 different layers, composed of convolutional layers, ReLu layers, pooling layers, and fully connected layers.

The work most similar to ours is the one proposed in [15], wherein the authors exploit the same dataset we used with a claimed lightweight CNN-2D. However, their analysis considered only the whole ECG image not partitioned per band as we did in [3], nor per single lead as conducted in this paper; they did not conduct any study on time or model complexity, thus the claimed lightweight feature of the proposed solution is rather questionable, nor did they consider the importance or influence of each single lead out of the twelve in terms of classification outcomes. Indeed, this could be particularly useful for the explainability of the obtained results even in the case of slightly worse performance. Differently from [15], in this paper, which is an extension of the work in [3] where we analyzed the three bands composing an ECG image, we performed an analysis considering the importance of each single lead in determining the classification outcome of different heart diseases as well as the impact of the signal of each lead on a particular disease. Moreover, we used a CNN-2D with fewer layers compared with the solution in [15] (eleven instead of twenty-two layers) and we obtained better results in identifying myocardial infarction (with recall values, which are the most important when considering medical data, often equal to 100%).

Starting from the studies already proposed in the literature, the approach we propose uses ECG images to extract the main features not only to identify the type of heart disease over time but also to intercept the particular electrode that characterizes it the most. In particular, concerning the characterization of the ECG track electrode per electrode, this is the first time as far as we know, that this analysis has been carried out.

## 3. Background

### 3.1. Convolutional Neural Networks

DL has flourished in many research areas in recent years, including image identification. In particular, CNNs are among the deep neural network variants that have received the best results in the analysis of images. Indeed, a CNN [16] is a feed-forward network that can easily process input signals, be they mono- or multi-dimensional. It is usually specialized for image processing and it is organized into many layers, every one of which emphasizes a distinct cognitive feature. CNN neurons, unlike neurons in a traditional neural network, are usually structured in three dimensions: width, height, and depth. The neurons in a layer, in particular, will not be completely linked to those in a close layer, making CNNs different from dense neural networks. For example, if the input image has the 180 × 180 × 3 (width, height, and depth, respectively) dimension, the final output layer will be 1 × 1 × 10 in size because the CNN will eventually condense the entire image into a single vector of class scores ordered along the depth axis. The CNN is normally composed of a sequence of ordered blocks, each of which is assigned to a network layer responsible for modulating the size of activation into another layer through the use of a differentiable function.

Hereunder, we summarize the main layers of a usual CNN [16]:

- The *Input layer*, which collects the initial data as a numeric array to be evaluated.
- The *Convolutional (Conv.) layers*, which use many filters to extract the key properties from the input data.
- The *Rectified Linear Units (ReLU) layers*, which are used to inject non-linearity into the network exploiting linear operations on the scalar product of the filters and the receptive field during the various convolutional layers. The outcome is an updated linearized feature map.
- The *Pool layers*, which rule the spatial scale of the convoluted features (height and breadth). Dimensionality reduction reduces the amount of computing power required to process the data. It is also useful for isolating dominant properties such as rotational and positional invariant components, enabling the network to be trained effectively.
- The *Fully Connected (FC) layers*, which have two principal aims: (i) connecting all the neurons of the previous layer to generate the various identification classes displayed in the prior layers depending on a certain probability, and (ii) returning the final classification. They are provided with an input volume, whatever the preceding convolutional layers, and generate a vector of size *N*, which corresponds to the number of classes from which the network must pick an outcome.

In particular, in this paper, we decided to use a CNN-2D architecture because this type of CNN proved to be capable of extracting important features from medical images in many situations [17], especially thanks to the aforementioned convolutional layers and pooling layers.

### 3.2. Cardiac Diseases

The heart is the basis of blood circulation. Its main function is to carry the blood, rich in oxygen, to the rest of the body, to cells, tissues, and organs. A healthy heart provides the appropriate blood volume for the entire body to continue functioning. Its several sections work in a coordinated manner at every instant of the living being’s life. When the heart is damaged or diseased, the organs cannot obtain the oxygen they require to work efficiently. Cardiopathy, which is a catch-all word for all types of heart diseases, is the interference with the pumping capacities of the heart. Furthermore, this issue can be hindered by structural difficulties, such as broken blood arteries, or functional problems, such as issues with the heart’s electrical system akin to arrhythmias.

This study analyzes two different types of cardiac disease:

- *Myocardial Infarction*, i.e., a myocardial lesion characterized by diagnostic evidence of acute myocardial ischemia and the detection of a rise or reduction in troponin cardiac values. Cardiac troponins “I” (cTnI) and “T” (cTnT) are myofibrillar proteins of the cardiac muscle involved in the phenomenon of contraction. These proteins are released by the cardiac muscle into the blood circulation during the contraction. Besides that, any of the symptoms listed in the following usually takes place: ECG changes, pathological Q-wave development, imaging evidence of new loss of viable myocardium, regional wall motion abnormality, and identification of a coronary thrombus through angiography or autopsy [18]. In particular, in this study, a distinction is made between ECGs of patients who have a myocardial infarction for the first time and patients who have had a previous history of myocardial infarction.
- *Abnormal Heartbeat*, also known as abnormal heart rate, which is an unusual number of times a person’s heart beats in one minute. The average heart rate varies from person to person. An adult’s heart should normally beat between 60 and 100 times every minute. Arrhythmia occurs when the heart pulses too slowly, too quickly, or irregularly. There are several kinds of cardiac arrhythmias: when the heartbeat rate falls below 60 beats per minute, and so the heart beats too slowly, this is called “bradycardia”; when the heartbeat rate exceeds 100 beats per minute and the heart beats faster, this is called “tachycardia”; in a generic arrhythmia, the rhythm of the beat rather than the speed is altered, as in the case of “extra-systole”, i.e., the existence of single or multiple unusual beats [19].

### 3.3. ECG Recording

The electrocardiogram is a diagnostic test that records and visualizes graphically the electrical activity of the myocardial fibers. It is simple to carry out and non-invasive; moreover, it allows the evaluation of the nature of a possible pathology, recognizing if it is mechanical or electrical. This examination can be performed at rest, with the patient lying supine to diagnose the presence of arrhythmias, ischemias and/or infarction, and acquired or congenital alterations of the heart cavities; or under the effort, while the patient walks on a treadmill or pedals a stationary bike, to evaluate the behavior of blood pressure, heart rate, and the possible appearance of signs or symptoms of cardiac distress during physical exertion. In our case study, the ECG is recorded at rest. In order to record the cardiac activity of the heart, some electrodes are applied to the skin of the patient; in particular, they are positioned on the limbs and the chest. The electrodes are connected, through electric wires, to an electrocardiograph that processes and prints the graphic tracing, i.e., the electrocardiogram, on paper. For the correct interpretation of the tracing, it is usually advisable to perform an ECG tracing at 12 derivations, to have twelve different points of view of the heart:

- *three derivations called “bi-polar limbs” (D-I, D-II, and D-III)*: three pairs of electrodes are used, i.e., the first pair on the right wrist and left wrist (D-I), the second on the right wrist and left ankle (D-II) and the third on the left wrist and left ankle (D-III), respectively. In particular, D-I records the potential between the left arm electrode (positive pole) and the right arm (red electrode), D-II records the potential between the left leg (positive pole) and right arm (negative pole), and D-III records the potential between the left leg (positive pole) and the left arm (negative pole);
- *three derivations called “uni-polar limbs” (aVR, aVL, and aVF)*: in uni-polar limb leads the positive electrode is connected to one of the limbs (usually the right leg), while the negative electrode is connected to a central terminal, which is approximately at zero potential. They are called: VF, which indicates the left foot, VR indicating the right arm, and VL, which indicates the left arm. The obtained values are amplified, so that they can be compared with those of the bipolar leads, thereby the actual values are indicated as aVR, aVL, and aVF, respectively;
- *six derivations called “pre-cordial leads” (V1, V2, V3, V4, V5, and V6)*: to conclude and to have a better definition of the overall cardiac activity, it is necessary to have electrodes that are close enough to the heart, as opposed to the previous ones which are far away. In particular, six electrodes are used to identify and localize, in a very precise way, lesions that could be missed with the use of other derivations.

The registration is made on graph paper, obtaining a printout of all 12 derivations. In general, paper scrolls at a speed of 25 mm/s, thus a small square of graph paper corresponds to 0.04 s (40 ms) and a large square corresponds to 0.2 s (200 ms) (Figure 1).

## 4. Approach

The used approach aims to classify cardiac disorders among three main categories: (i) those with myocardial abnormalities (MI), (ii) those with a past myocardial infarction (PMI), and (iii) those with arrhythmias (HB).

**Figure 2:**
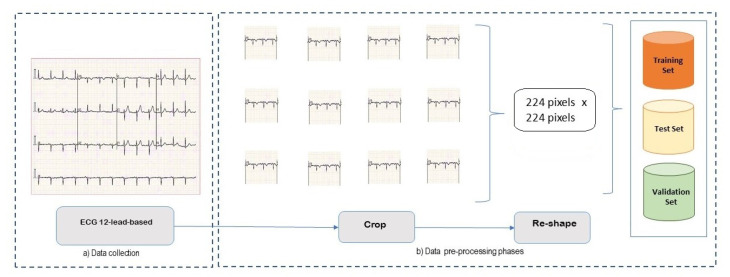
Details of the used approach: (**a**) data collection and (**b**) pre-processing.

**Figure 3:**
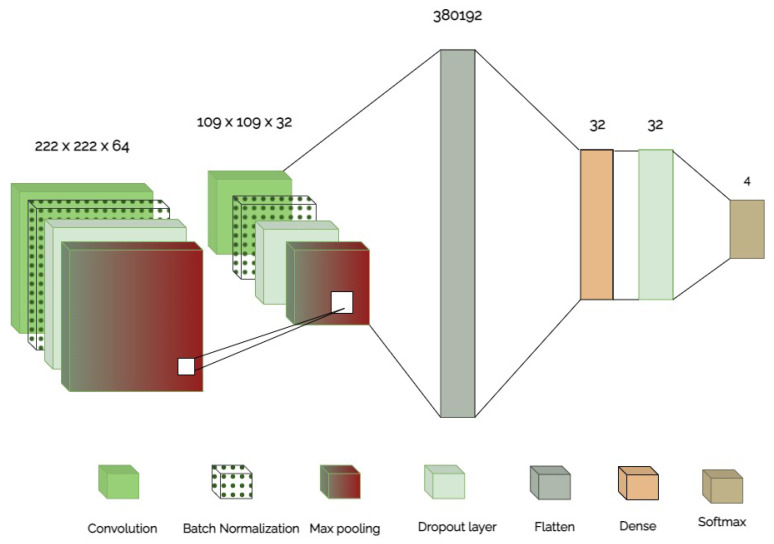
CNN-2D architecture used for the classification of ECG images per single lead.

Figure 2 depicts the details of the data collection and of the pre-processing phase. In particular, Figure 2a depicts the ECG gathering phase, which exploits data from an existing dataset [20], wherein ECG images (in *.jpg* format) of patients are acquired from the 12 different sensors as described in Section 3.3. Figure 2b describes the steps necessary for the next phase regarding the generation of 12 new datasets, one for each lead of the ECG: the part of the image depicting every single electrode is first segmented; later the cropped images were reshaped because they had different pixel dimensions and too many pixels, which could lead to a long time to train and test the neural network models. As a result of these two pre-processing procedures, the resulting datasets were segmented into train, test, and validation sets (split ratio: 60/20/20). Finally, Figure 3 outlines the structure of the CNN-2D under consideration. The case study is built on a CNN-2D for each specific ECG lead for the four classes of patients (three categories of ill patients and healthy subjects). The input layer is followed by more hidden layers providing convolutional, batch normalization, max pooling, dropout, as well as flattening operations, and an output layer that returns the result of the image classification for each single lead.

### 4.1. Dataset

The data used in this empirical study are publicly viewable [20] and consist of ECG pictures of cardiac patients obtained from the Ch. Pervaiz Elahi Institute of Cardiology Multan (Pakistan), which intends to promote research in the study of cardiovascular disorders. ECG recordings from 928 participants are included in the dataset. All of the ECG pictures were obtained by using 12 leads, whose electrical signals were captured using the Telehealth ECG screening tool, and they are greater than 800 KB individually.

The original images have been split into four different classes:

- *Group of myocardial infarction patients* (MI): referring to the necrosis of a portion of the heart muscle caused by the blockage of one or more coronary arteries; this collection is made up of 239 ECG pictures.
- *Group of Patients that have abnormal heartbeat* (HB): this group is related to patients with arrhythmias and is composed of 233 ECG images.
- *Group of patients that have a history of MI* (PMI): this class referred to 172 ECG images of patients with a past myocardial infarction.
- *Group of health controls* (Normal): this class collected the ECG of 284 subjects with no cardiac disease at all.

### 4.2. Setting of CNN-2D

The CNN-2D model used to categorize the ECG is detailed in Table 1, which outlines the model setup. In particular, the first column of the table identifies the level, the second column defines the kind of layer used in the reference level, the third column shows the pattern of the output generated from that level, and the last column indicates the kernel size where necessary.

The additional parameters of the CNN-2D network model are the following:

1. *Activation function*: the activation function is an essential element in the design of a neural network because it participates in the transformation of the input into the output. We use the Rectified Linear Unit activation function (ReLU) [21] via *‘relu activation’* for its simplicity of calculation, representational sparsity, and because it is similar to the linear activation function (it has the same performance and actions) and this optimizes the behavior of the neural network;
2. *Optimizer*: Choosing the optimization algorithm in a deep learning model can make the difference between good results in minutes, hours, and days. In this experimentation, the Stochastic Gradient Descent (SGD) is replaced by *Adam* [22] because it is faster to implement, uses a smaller number of tuning parameters, is computationally efficient, and has restricted memory requirements;
3. *Dropout rate*: it is typically used to increase network adaptability by decreasing the over-fitting of the networks. In this study, the dropout rate was set to 0.15.

The architectures of the convolutional neural network were implemented using Tensorflow (https://www.tensorflow.org/), Keras (https://keras.io/), and the Python programming language. The first is an open-source software framework for machine learning and Artificial Intelligence that can be employed for a wide range of purposes, with a special emphasis on deep neural network training as well as inference. Keras is a free software program, which supplies a user-friendly Tensorflow library interface and offers a Python API for artificial neural networks.

Finally, the evaluated model was trained on 100 epochs and a batch size equal to 32.

### 4.3. Validation

In order to validate the proposed approach, the confusion matrix is used to determine the classification results and to measure the following validation performance measures: true positives (TP) and true negatives (TN), i.e., correctly classified instances; false positives (FP) and false negatives (FN), i.e., incorrectly identified instances. These elements are crucial for calculating the following metrics:

- *Accuracy (A)*, an overall metric that evaluates the total proportion of correct predictions:
  (1) $$A=\frac{TP+TN}{TP+FP+TN+FN}$$
- *Precision (P)*, calculating the proportion of positive correct predictions (TP) over the overall positive predictions made by the network:
  (2) $$P=\frac{TP}{TP+FP}$$
- *Recall (R)*, also called sensitivity, which is the ratio between the correct positive predictions (TP) over the total number of relevant instances to predict:
  (3) $$R=\frac{TP}{TP+FN}$$
- *F1-Score (F1)*, which is the harmonic mean between precision and recall:
  (4) $$F1=2\cdot \frac{P\cdot R}{P+R}.$$

These metrics are the most used in the validation of a classification model; in particular, in medical data validation, recall is greatly emphasized as a crucial metric because it prioritizes the identification of actual positive cases, which is vital for ensuring patient safety and effective treatment. A good value of recall is particularly important in medical applications because failing to identify positive cases (false negatives) can lead to missed diagnoses or delayed treatments, potentially resulting in severe health consequences. However, while recall is a critical metric, it must be interpreted in conjunction with other evaluation metrics to provide a comprehensive assessment of the model’s performance. This multiple metrics approach helps ensure that the model is not only identifying the majority of positive cases but also maintaining an overall acceptable level of accuracy and precision.

In parallel to these criteria, the *loss function* was computed: it is defined as the variation between the actual output and the output that the model forecasts and it contributes to improving the model’s performance. In the proposed study, due to the nature of the data, we employed the *categorical cross-entropy* like a probability-based loss function. Lastly, while training, the accuracy and loss function were investigated for each epoch and explored for all network configurations.

## 5. Results

Table 2 reports the global metrics of validation of the classification on the single electrode of an ECG. In particular, the first column reports the name of the electrode, the second column the global accuracy, the third column the precision metric, the fourth column the results of the recall metric, and the last column the F1-score. Since we were evaluating medical data referring to a disease, it was more appropriate to evaluate the classifications through the *Recall metric* because it is really important to avoid false negatives as explained in Section 4.3. Since the analyses were carried out on real ECG data, we have assumed that the values of Recall ≥80% can be considered acceptable as it usually happens whenever using real medical data. The highest recall results per electrode are highlighted in bold in Table 2. In particular, we found that the best classifications were those related to the electrodes (in order from the highest to the lowest value of Recall) D-II (89%), V3 and V5 (87%), aVF (86%), aVR (85%), and D-I (81%). Furthermore, Table 3 reports the results referred to the classification of the three heart diseases based on the CNN-2D applied to the single image of an electrode. In particular, the first column reports the type of heart-related disease, and the columns from the second one onward report the evaluation metrics for the analyses carried out on the single electrodes.

Also, in Table 3 it is preferred to use the *Recall metric* to understand which are the best electrodes to characterize the various cardiopathies. The results written in bold are those related to the highest values of Recall. They show that myocardial infarction is the best-classified cardiopathy, given that most of the highest Recall values are linked with the MI class of patients, i.e., eleven electrodes have Recall ≥80%).

The results of the MI class show that the electrodes that best characterize this pathology are the three bipolar leads, which take into account the tracing of the peripheral limbs, and the six pre-cordial leads, which detect the tracing closest to the heart since these electrodes are placed on the patient’s back.

The second best-ranked heart disease (eight electrodes having Recall ≥80%) is related to a prior history of myocardial infarction. The study of the eight best-classified electrodes for patients with PMI shows that the electrodes that best characterize this pathology are those related to the three unipolar leads, which take into account the potential tracing of the peripheral limbs.

The heart disease that returns the most false negatives is the one connected to irregular heartbeat or arrhythmias (HB). This pathology is the most difficult to recognize and characterize in the per-lead analysis we carried out.

Furthermore, we tried to evaluate the performance of the cardiac disorder detection system by contrasting it with previous studies focused on ECG cardiac classification, but it is not very feasible because of two main reasons:

- it is essential to note that several referenced works utilize a distinct dataset involving time-series data and diverse class categories, making a direct comparison of the results infeasible;
- the work that uses the same dataset as our study, as already stated in Section 2, never studied the single electrode approach, thus also this comparison is poorly meaningful in terms of overall performance. However, the work in Section 2 used a more complex CNN-2D architecture compared with ours.

Despite these differences, our study demonstrates notable success in detecting three cardiac abnormalities and a very novel approach, based on single lead characterization, for the interpretable classification of the ECGs.

## 6. Discussion

This study demonstrates the validity of the used approach and allows diversifying the reading of the 12 electrodes used for ECG recording to obtain a more meaningful and explainable diagnosis.

This approach could result in useful support for doctors in the diagnostic process of myocardial infarction and arrhythmias since the interpretation of the ECG is performed on single electrodes, effectively improving the diagnosis. Therefore, the doctors could pay attention to a single electrode or to a specific group of electrodes based on the type of disease to be recognized and not to the entire ECG. In particular, this approach is functional in discriminating against atrioventricular disorders such as myocardial ischemia.

Indeed, the analyses showed some interesting evidence:

- it is possible to recognize myocardial necrosis by paying more attention to the bipolar electrodes;
- to better identify a heart that suffers from myocardial infarction, it is necessary to pay attention to the uni-polar leads of the ECG;
- the generic arrhythmias of the heartbeat are more laborious to recognize through the study of the single electrode because the identification of the arrhythmia is based on a global interpretation of the ECG rather than on individual electrodes.

At the moment it is not possible to compare this work with many other previous works, except for what already said in Section 2 about the paper in [15], because the traditional literature heretofore uses the whole ECG, mainly as an electric signal, to classify heart disease and not the single electrode. Conversely, the proposed method exploits ECG images to extract features concerning the single electrode and, to the best of our knowledge, this is the first time that a similar approach has been used. Furthermore, although this is a preliminary analysis of this methodology, the results are newsworthy and show the efficiency of the approach itself.

The approach used for ECG classification can be easily applied in medicine to help and improve the interpretation of ECGs by cardiologists because it can classify ECG images in almost real-time and it does not require long processing times and expensive equipment, once the CNN has been properly trained. For these reasons, it could play an important role in future digital healthcare systems.

Some limitations of this study concern the size of our dataset, which may restrict the generalizability of the results. Indeed, in this study, we have focused on a specific dataset and we are aware that the findings described in this study could be only consolidated and generalized in our future work using other ECG image datasets, possibly entailing other heart-related pathologies.

## 7. Conclusions

This study suggests a CNN-2D technique for functional ECG classification, using ECG images as input. We separated the original input ECG image into 12 separate images, one for each ECG electrode, to characterize the ECG classification with higher quality and interpretability.

We classified the ECG images into three groups of heart diseases, obtaining in some cases recall values close to or exactly equal to 100%, and identifying the most important electrodes for the classification of certain diseases as well.

For future work, the architecture of the used CNN-2D will be further optimized to obtain better performance results and more ECG images possibly will be collected to enhance the training and test datasets. Moreover, the proposed solution will be compared with other ML and DL techniques, providing explainable rules as well, and we will also target different and more subtle heart diseases. Furthermore, we will evaluate the complexity and feasibility of the proposed solution for a real-world practical implementation in the field.

## Figures and Tables

**Table 1:** Configuration of the used CNN-2D.

Layer	Type	Output Shape	Kernel Size
I	Convolution	(222, 222, 64)	3
II	Batch Normalization	(222, 222, 64)	-
III	Drop Out	(222, 222, 64)	-
IV	Pooling	(111, 111, 64)	2
V	Convolution	(109, 109, 32)	3
VI	Batch Normalization	(109, 109, 32)	-
VII	Drop Out	(109, 109, 32)	-
VIII	Flatten	(380192)	-
IX	Dense	32	-
X	Drop Out	32	-
XI	Sofmax	4	-

**Table 2:** Global metrics of classification on the single electrode of an ECG.

Electrode	Accuracy	Precision	Recall	F1-Score
**D-I**	0.80	0.79	**0.81**	0.79
**D-II**	0.88	0.87	**0.89**	0.88
D-III	0.80	0.81	0.78	0.79
**aVF**	0.83	0.84	**0.86**	0.84
aVL	0.73	0.80	0.76	0.74
**aVR**	0.81	0.84	**0.85**	0.82
V1	0.78	0.79	0.79	0.77
**V2**	0.78	0.80	**0.82**	0.78
**V3**	0.84	0.85	**0.87**	0.84
V4	0.78	079	0.81	0.77
**V5**	0.88	0.87	**0.87**	0.87
V6	0.73	0.75	0.76	0.73

**Table 3:** Metrics of validation for single electrode per class of disease.

	Electrode								
	**D-I**			**D-II**			**D-III**		
**HEART DISEASE**	**PRECISION**	**RECALL**	**F1-SCORE**	**PRECISION**	**RECALL**	**F1-SCORE**	**PRECISION**	**RECALL**	**F1-SCORE**
**HB**	0.89	0.70	0.78	0.98	**0.81**	0.88	0.76	**0.88**	0.81
**MI**	0.77	**1.00**	0.87	0.91	**1.00**	0.95	0.94	**0.80**	0.86
**PMI**	0.72	0.72	0.72	0.74	**0.88**	0.88	0.72	0.66	0.69
	**aVF**			**aVL**			**aVR**		
**HEART DISEASE**	**PRECISION**	**RECALL**	**F1-SCORE**	**PRECISION**	**RECALL**	**F1-SCORE**	**PRECISION**	**RECALL**	**F1-SCORE**
**HB**	0.93	0.68	0.79	0.92	0.60	0.72	1.00	0.67	0.80
**MI**	0.91	**1.00**	0.95	1.00	0.72	0.84	0.95	**0.90**	0.92
**PMI**	0.67	**0.91**	0.77	0.49	**0.97**	0.65	0.58	**0.97**	0.73
	**V1**			**V2**			**V3**		
**HEART DISEASE**	**PRECISION**	**RECALL**	**F1-SCORE**	**PRECISION**	**RECALL**	**F1-SCORE**	**PRECISION**	**RECALL**	**F1-SCORE**
**HB**	0.88	0.65	0.75	0.97	0.56	0.71	1.00	0.65	0.79
**MI**	0.97	**0.88**	0.92	0.77	**1.00**	0.87	0.78	**1.00**	0.88
**PMI**	0.53	**0.84**	0.65	0.66	**0.91**	0.75	0.76	**0.97**	0.85
	**V4**			**V5**			**V6**		
**HEART DISEASE**	**PRECISION**	**RECALL**	**F1-SCORE**	**PRECISION**	**RECALL**	**F1-SCORE**	**PRECISION**	**RECALL**	**F1-SCORE**
**HB**	0.97	0.56	0.71	0.89	**0.84**	0.86	0.91	0.51	0.65
**MI**	0.71	**1.00**	0.83	0.91	**1.00**	0.95	0.66	**1.00**	0.79
**PMI**	0.70	**0.88**	0.78	0.81	0.78	0.79	0.69	0.78	0.74

## Data Availability

Data are contained within the article.
